# Satisfaction with the Exercise Program and Successful Aging Among Older Adults Who Exercise Regularly: The Multiple Mediation of Physical Self-Efficacy and Exercise Adherence

**DOI:** 10.3390/healthcare12202054

**Published:** 2024-10-16

**Authors:** Hyun-Ryun Kim, Seung-Hwan Woo, Jae-Pil Seo, Wi-Young So, Jun-Su Bae

**Affiliations:** 1 Department of Physical Education, Woosuk University, Wanju-gun 55338, Jeollabuk-do, Republic of Korea; khr870615@woosuk.ac.kr; 2 College of Convergence in Culture, U1 University, Yeongdong-eup 29131, Chungcheongbuk-do, Republic of Korea; woosh0916@u1.ac.kr; 3 Sports & Leisure Studies, Gimcheon University, Gimcheon-si 39528, Gyeongsangbuk-do, Republic of Korea; 0025@gimcheon.ac.kr; 4 Department of Sports Medicine, College of Humanities, Korea National University of Transportation, Chungju-si 27469, Chungcheongbuk-do, Republic of Korea; 5 Institution of Sport Contents, Andong National University, Anndong-si 36729, Gyeongsangbuk-do, Republic of Korea

**Keywords:** aging society, exercise psychology, health promotion, lifestyle, public health

## Abstract

Objectives: As the Republic of Korea transitions towards a super-aged society, the health and mental well-being of older adults are emerging as critical issues. While many researchers are focusing on successful aging among older adults who participate in exercise, there is a research gap in the Korean literature regarding the preconditions that foster this outcome. Therefore, this study analyzed the relationship between satisfaction and exercise, physical self-efficacy (PSE), exercise adherence, and successful aging among Korean older adults who exercise regularly. Methods: The participants were 369 (234 male and 135 female) older adults aged 65 or older living in the Republic of Korea, with an average age of 69.91 years. Data were collected from March 2024 to June 2024 and analyzed using descriptive statistics, correlation analysis, and structural equation modeling. Results: There was no problem with the model fit. Satisfaction with exercise positively affected PSE (β = 0.317, *p* < 0.001), exercise adherence (β = 0.487, *p* < 0.001), and successful aging (β = 0.669, *p* < 0.001). PSE positively affected exercise adherence (β = 0.356, *p* < 0.001), and exercise adherence positively affected successful aging (β = 0.190, *p* < 0.05). Furthermore, PSE (β = 0.019, *p* < 0.01) and exercise adherence (β = 0.081, *p* < 0.05) mediated the relationship between satisfaction with exercise and successful aging. Conclusions: Satisfaction with exercise is important for promoting successful aging among Korean older adults who exercise. Satisfaction with exercise can increase their efficacy for performing physical activities and encourage them to continue exercising. This can ultimately lead to a happy life in old age.

## 1. Introduction

Experts have predicted that by 2025, South Korea will become a super-aged society, with individuals aged 65 or older exceeding 20% of the population. However, a more serious concern is the accelerated pace of aging, as reports have predicted that by 2060, older adults will make up 40% of the country’s population [1]. Aging is considered a universal and natural phenomenon. However, a large proportion of older adults in society is linked to various social problems, such as increased social costs, decreased productivity, and inter-generational conflicts [2]. Moreover, older adults also face an array of problems, such as lethargy due to the loss of roles, the inability to participate in social activities due to health problems, and increased medical costs [3]. In this way, an aging society has more drawbacks than benefits, not only for society but also for older adults, necessitating its addressal.

The Korean government has implemented several policies to cope with the problems of an aging society. However, these efforts have been unable to match the pace of societal change. Researchers have advocated for more fundamental solutions, one of which is the promotion of older adults’ well-being. A concept analogous to older adults’ well-being is successful aging. Successful aging is a concept introduced by Rowe and Kahn [4], who compared older adults who experienced usual aging with those who did not experience usual aging and sought to identify the characteristics of individuals who continue to live happily in their old age. Since then, researchers focusing on successful aging have proposed various theoretical models, and successful aging has become a salient concept among scholars studying old age [5,6]. Successful aging has three characteristics: (1) low risk of health-related problems, such as injury, disease, or disability; (2) relatively high levels of physical and cognitive functioning; and (3) a proactive approach toward life, even after professional retirement [7]. Therefore, successful aging is a multidimensional concept encompassing physical, emotional, cognitive, and social aspects [8].

Previous research has shown that exercise participation promotes successful aging [9,10,11,12]. For example, Chou et al. [13] analyzed several studies and reported that exercise improves older adults’ walking speed, balance, and activities of daily living. Liberman et al. [14] found that exercise is effective for muscle strength, body composition, physical function, and inflammation. Hou et al. [15] reported that exercise has a positive effect on older adults’ mental health. Thus, it can be considered that these physical and psychological effects contribute to successful aging. However, studies have also shown that it is not short-term exercise participation that is beneficial; rather, it is long-term and regular exercise participation. In other words, exercise adherence is required for successful aging.

Exercise adherence refers to the level of one’s exercise engagement and participation. It indicates one’s attachment to and obsession with the exercise one participates in and a state in which there is no intention of dropping out [16]. Individuals with high levels of exercise adherence consider exercise a part of their lives [17]. In fact, a longitudinal study spanning over six years [18] found that older adults who consistently exercised and actively engaged in various activities reported more positive health indicators related to successful aging, including mental well-being, lower mortality rates, and productive activities. Additionally, many previous studies have provided evidence that regular participation in exercise is directly related to successful aging among older adults [19,20]. However, older adults face numerous difficulties in continuing exercise, such as family problems, financial problems, interpersonal relationship problems, and skepticism about life [21]. The most notable obstacle is the subjective evaluation of one’s physical ability. Because exercise requires physical ability, how one perceives one’s physical ability has a significant impact on whether one would continue to exercise. If an older individual believes that their physical ability is satisfactory, they will likely continue exercising. Conversely, if they consider their physical ability unsatisfactory, they may avoid exercising. This subjective evaluation of one’s physical ability is known as physical self-efficacy (PSE) [22].

PSE refers to the belief that one can successfully complete a physical activity in a specific or diverse situation [23]. The concept of PSE was developed based on Bandura’s [24] social cognitive theory and has characteristics similar to those of general self-efficacy. Bandura [25] argued that PSE affects one’s participation in physical activity and decision-making concerning it. Individuals with high levels of PSE invest more effort in overcoming the obstacles and difficulties they face [25]. Therefore, it can be predicted that the higher the PSE in older adults, the greater their willingness to participate in and continue exercising. In fact, Warner et al. [26] found that self-efficacy for exercise (or PSE) is related to the frequency of exercise participation among older adults. This supports the fact that PSE predicts the continuation of exercise among older individuals.

Among older individuals who exercise regularly, their PSE, exercise adherence, and successful aging come from their satisfaction with exercise. Satisfaction with exercise is the level of satisfaction one feels from participating in exercise [27]. Whether one is satisfied with the health changes one experiences due to exercise indicates one’s physical satisfaction. Whether one is satisfied with the relationships one builds due to exercise indicates one’s social satisfaction. Whether one is satisfied with the changes in one’s cognition or way of thinking indicates one’s psychological satisfaction. Studies have shown that one’s satisfaction with exercise can predict one’s self-efficacy, exercise adherence, and successful aging [28,29,30]. Older individuals who experience high levels of physical, social, and psychological satisfaction from participating in exercise tend to experience more positive emotions, and this positive psychological state enhances PSE. Furthermore, older adults who perceive an improvement in PSE are more likely to engage in long-term exercise participation, ultimately enabling them to meet the conditions necessary for successful aging.

**Figure 1:**
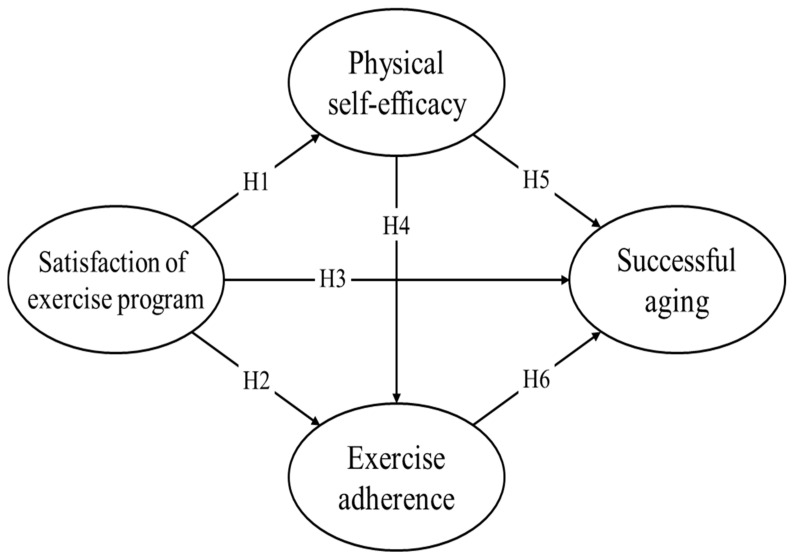
Hypothesized model. Note: H7 is an indirect path through H1 and H5; H8 is an indirect path through H2 and H6; H9 is an indirect path through H1, H4, and H6.

However, previous studies have only provided indirect evidence regarding the relationships among exercise satisfaction, PSE, exercise adherence, and successful aging, and very few studies have empirically verified decisive evidence for these theoretical relationships. Furthermore, it is necessary to examine the specific processes through which exercise satisfaction influences successful aging via PSE and exercise adherence. By pursuing this endeavor, we can expand our understanding of older individuals’ participation in exercise and successful aging. Most importantly, this research can contribute to the development of the relatively underexplored literature on this topic in the Korean context. Considering that South Korea may soon become a super-aged society, it is important to focus on the healthy and happy lives of Korean older adults. However, research on their satisfaction with exercise participation and related variables is lacking. More specifically, there is a lack of research on the theoretical structure of how satisfaction with exercise is related to PSE, exercise adherence, and successful aging among Korean older adults who exercise regularly. Such research is expected to promote exercise participation among Korean older adults and provide useful information to experts and policymakers. Therefore, this study aimed to verify the structural relationship between satisfaction with exercise, PSE, exercise adherence, and successful aging among Korean older adults who regularly participate in sports activities. To achieve the research objectives, nine hypotheses were established. The research model is depicted in Figure 1.

**H1:**  *Exercise satisfaction will influence PSE*.

**H2:**  *Exercise satisfaction will influence exercise adherence*.

**H3:**  *Exercise satisfaction will influence successful aging*.

**H4:**  *PSE will influence exercise adherence*.

**H5:**  *PSE will influence successful aging*.

**H6:**  *Exercise adherence will influence successful aging*.

**H7:**  *Exercise satisfaction will affect successful aging through the mediation of PSE*.

**H8:**  *Exercise satisfaction will affect successful aging through the mediation of exercise adherence*.

**H9:**  *Exercise satisfaction will influence successful aging through the mediation of both PSE and exercise adherence*.

## 2. Materials and Methods

### 2.1. Participants

The duration of exercise participation among older individuals can significantly interfere with research outcomes. For example, older adults who have participated in exercise for a shorter period may exhibit considerable personal variability in levels of exercise satisfaction, PSE, and exercise adherence due to their relatively recent engagement. It can be particularly anticipated that there will be significant differences in exercise adherence based on the duration of participation. Since this study focuses on verifying the structural relationships among the research variables rather than examining the differences based on duration, it is essential to minimize the interference of exercise participation duration. To achieve this, the selected participants were adults aged 65 years and older who have been engaged in one or more types of physical activity or sports on a long-term basis. Although the criteria for long-term exercise participation are not clearly defined, a duration of one year was established for the present study through discussions among the researchers.

A total of 396 participants were recruited. However, 27 participants were excluded owing to insincere or missing data. The sample size recommended by the power analysis was 119 participants and was calculated using the G* Power software (G* Power 3.1.9.7, Hein-rich-Heine-University, Düsseldorf, Germany). Consequently, we used the responses of 369 participants. The participants’ average age was 69.91 years (standard deviation [SD] = 3.84, range = 65–82 years). As shown in Table 1, most participants were male (63.41%). Individuals who participated in hiking were the highest in number (27.37%), followed by those who participated in swimming (13.28%), cycling (11.65%), table tennis (8.13%), badminton (8.13%), and tennis (7.59%). On average, the participants had participated in their respective physical activities for 15.00 years (SD = 3.07, range = 3~31). They participated in their respective physical activities 3.40 days a week (SD = 1.41) for 2.52 h per session (SD = 0.89) on average.

### 2.2. Data Collection

This study was conducted in accordance with the principles outlined in the Declaration of Helsinki, and the study protocol was approved by the Institutional Review Board of Woosuk University (IRB No. WS-2023-41). After obtaining IRB approval, we sought cooperation from several senior sports clubs and program managers at city halls in the metropolitan areas of Seoul, Gyeonggi, Chungcheongnam-do, and Jeollabuk-do, Republic of Korea, to recruit participants. The researchers visited institutions that had cooperated in the past and posted recruitment notices at those institutions. Only individuals aged 65 years or older with a voluntary willingness to participate were selected for the study. A consent form and questionnaire were distributed to the participants. At that time, the researchers explained the contents of the consent form and how to complete the questionnaire. They also explained about data processing methods and research ethics, such as the Personal Information Protection Act. The participants completed and submitted the questionnaire on the spot. To increase the sincerity of responses, the researchers provided a small gift (e.g., simple sports equipment) to the participants. Data were collected at 17 institutions from March 2024 to June 2024.

### 2.3. Measures

The survey tool of this study was a structured questionnaire. All variables and questions were consistent with the purpose of this study but were constructed based on a questionnaire whose validity and reliability have been verified in previous studies. Specifically, satisfaction with exercise was assessed using Lee’s [31] questionnaire, which measures one’s subjective satisfaction with participation in recreational sports. The questionnaire contained 9 items measuring physical satisfaction (3 items), social satisfaction (3 items), and psychological satisfaction (3 items). All items were rated on a 5-point Likert scale ranging from 1 (“strongly disagree”) to 5 (“strongly agree”).

The Exercise Adherence Rating Scale developed by Newman-Beinart et al. [32] was adapted to the context of Korean older adults to assess exercise adherence in this study. The scale consisted of a single factor and six items on exercise practice. All items were rated on a 5-point Likert scale ranging from 1 (“strongly disagree”) to 5 (“strongly agree”).

PSE was measured using the Korean version [33] of the PSE Scale, which was developed by Ryckman et al. [22]. This questionnaire measures one’s efficacy for performing physical activities or expressing oneself through one’s body. It comprised 22 items, with 10 items on Perceived Physical Ability (PPA) and 12 items on Physical Self-Presentation Confidence (PSPC). All items are rated on a 5-point Likert scale ranging from 1 (“strongly disagree”) to 5 (“strongly agree”).

Successful aging was measured using the Korean Elderly’s Successful Aging Scale [34], which was developed based on Ryff’s [6] theory. This scale comprised 31 items across six factors: autonomous life (AL; 9 items), self-completion orientation (SCO; 6 items), positive life participation (PLP; 5 items), satisfaction with one’s offspring (SWO; 5 items), self-acceptance (SA; 3 items), and acceptance of others (OA; 3 items). All items were rated on a 5-point Likert scale ranging from 1 (“strongly disagree”) to 5 (“strongly agree”).

### 2.4. Data Analysis

Before using the constructed questionnaire, its content validity was verified by a group of experts (one professor majoring in physical education, one professor majoring in psychology, and one senior sports instructor). Then, we performed confirmatory factor analysis (CFA) using the maximum likelihood estimation method, along with a reliability analysis using Cronbach’s α, to determine the construct validity and reliability of the measurement tools used. The data collected were analyzed in the following manner: First, descriptive statistics were calculated to determine the tendency and normality of data. The mean and SD were calculated for each item to check whether there were any abnormalities in the collected data. Then, skewness and kurtosis were calculated to examine the assumption of normality. Following Kline [35], if the skewness value was 3 or less and the kurtosis value was 8 or less, the data was considered to be normal. Second, a bivariate correlation analysis was performed using Pearson’s product–moment correlation coefficient. This was done to determine the relationship between the variables. Multicollinearity was examined using correlation analysis. Third, structural equation modeling was performed to verify the research model. The indices for judging the model’s adequacy were *x*^2^/*df*, CFI, TLI, RMSEA, and SRMR, which are commonly used in social science research. Model fit was considered if the *x*^2^/*df* was 3 or less, CFI and TLI were 0.900 or higher, and RMSEA and SRMR were 0.080 or less [35]. Finally, a phantom model analysis was performed to verify multiple mediations. In this analysis, phantom variables are created to verify the significance of the mediating effect of each variable when there are two or more mediating variables [36]. To verify the mediation effect, bootstrapping was performed 1000 times, and the estimation of the confidence interval (CI) was set to the 95% CI level of the bias-corrected method. If zero was not included in the CI, the mediation effect was considered significant. Statistical significance was set at *p <* 0.05. All statistical analyses were performed using SPSS and AMOS for Windows (version 24.0; IBM Corp., Armonk, NY, USA).

## 3. Results

### 3.1. Validity and Reliability of the Measurement Tools

#### 3.1.1. Satisfaction with Exercise

The CFA showed that the model fit indices were *x*^2^/*df* = 2.978 (*x*^2^ = 71.464, *df* = 24, *p* < 0.001), comparative fit index (CFI) = 0.970, Tucker–Lewis index (TLI) = 0.955, standardized root mean square residual (SRMR) = 0.042, and root mean square error of approximation (RMSEA) = 0.073 (95% CI = 0.054–0.093). The Cronbach’s α coefficient was 0.880, 0.793, and 0.785 for physical, social, and psychological satisfaction, respectively (see Table 2).

#### 3.1.2. Exercise Adherence

The CFA showed that the model fit indices were *x*^2^/*df* = 2.912 (*x*^2^ = 26.212, *df* = 9, *p* < 0.001), CFI = 0.981, TLI = 0.969, SRMR = 0.028, and RMSEA = 0.072 (95% CI = 0.041–0.105). Reliability was confirmed with a Cronbach’s α coefficient of 0.866 (see Table 3). We parceled exercise adherence into three measurement variables for data analysis. Parceling items were randomly selected in groups of two.

#### 3.1.3. PSE

The CFA showed that the model fit indices were *x*^2^/*df* = 3.066 (*x*^2^ = 628.437, *df* = 205, *p* < 0.001), CFI = 0.912, TLI = 0.901, SRMR = 0.074, and RMSEA = 0.075 (95% CI = 0.060–0.082). Reliability was confirmed with Cronbach’s α coefficients of 0.928 and 0.918 for PPA and PSPC, respectively (see Table 4).

#### 3.1.4. Successful Aging

During the analysis of the validity of the measurement tool, the loading of one item under self-completion orientation (“I donate material things to others when I have the chance”) was insignificant; thus, it was removed. The CFA results showed that the model fit indices were *x*^2^/*df* = 2.430 (*x*^2^ = 930.680, *df* = 383, *p* < 0.001), CFI = 0.902, TLI = 0.888, SRMR = 0.052, and RMSEA = 0.062 (95% CI = 0.057–0.067). Reliability was confirmed with Cronbach’s α coefficients of 0.820, 0.652, 0.869, 0.838, 0.842, and 0.825 for AL, SCO, PLP, SWO, SA, and OA, respectively (see Table 5).

### 3.2. Descriptive Statistics and Correlation Coefficients

Table 6 shows descriptive statistics and bivariate correlation coefficients of the variables. The mean of the variables ranged from 3.445 to 4.364. The SD ranged from 0.484 to 0.813. The skewness and kurtosis values showed that the collected data met normality standards. The bivariate correlation analysis showed that most sub-factors were significantly and positively correlated (*p* < 0.001). Only the correlation between PPA and satisfaction with one’s offspring was not statistically significant (*p* = 0.085). The remaining bivariate correlation coefficients ranged from 0.167 to 0.474. Furthermore, there were no concerns of multicollinearity stemming from a high correlation between variables.

### 3.3. Structural Equation Modeling

Before the hypotheses were verified, the fit of the research model was examined. An analysis of the model fit showed *x*^2^/*df* = 2.739 (*x*^2^ = 194.494, *df* = 71, *p* < 0.001), CFI = 0.943, TLI = 0.927, SRMR = 0.056, and RMSEA = 0.069 (95% CI = 0.057–0.080). These indices demonstrated that the research model had a “very good fit”. Additionally, to further examine multicollinearity, a CFA was conducted on the model, and the correlations between latent variables were assessed. The results indicated that the correlation coefficients between latent variables ranged from 0.201 to 0.609, suggesting that there is no issue regarding multicollinearity.

#### 3.3.1. Direct Paths

**Figure 2:**
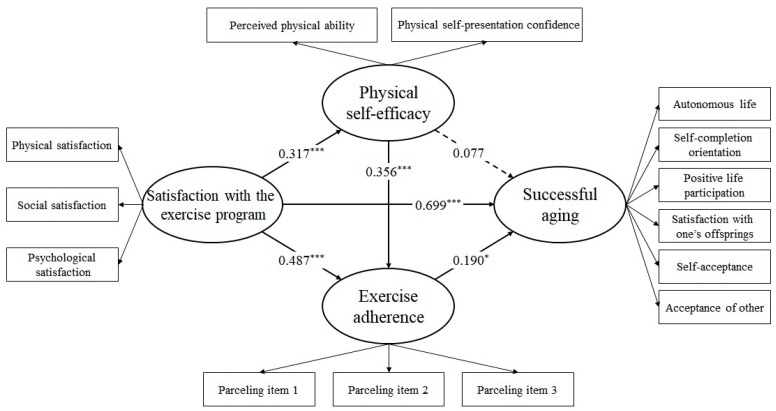
Standardized estimates in the research model (* *p* < 0.05, *** *p* < 0.001). PPA, Perceived Physical Ability; PSPC, Physical Self-Presentation Confidence.

Table 7 presents the results of examining direct paths. Five of the six direct paths were statistically significant (*p* < 0.05), and one path was not (*p* > 0.05). Satisfaction with exercise positively affected PSE, with a standardized regression coefficient of 0.317 (*t* = 4.614, *p* < 0.001). It also had a positive effect on exercise adherence, with a standardized regression coefficient of 0.487 (*t* = 7.067, *p* < 0.001). PSE positively affected exercise adherence, with a standardized regression coefficient of 0.356 (*t* = 4.435, *p* < 0.001). However, it did not have a statistically significant effect on successful aging (*p* > 0.05). Exercise adherence positively affected successful aging, with a standardized regression coefficient of 0.190 (*t* = 2.158, *p* < 0.05). Finally, satisfaction with exercise positively affected successful aging, with a standardized regression coefficient of 0.699 (*t* = 7.218, *p* < 0.001). Figure 2 shows the standardized regression coefficients of direct paths in the research model. Statistically significant paths are indicated by solid lines, while statistically insignificant paths are indicated by dotted lines in the figure.

#### 3.3.2. Indirect Paths

**Figure 3:**
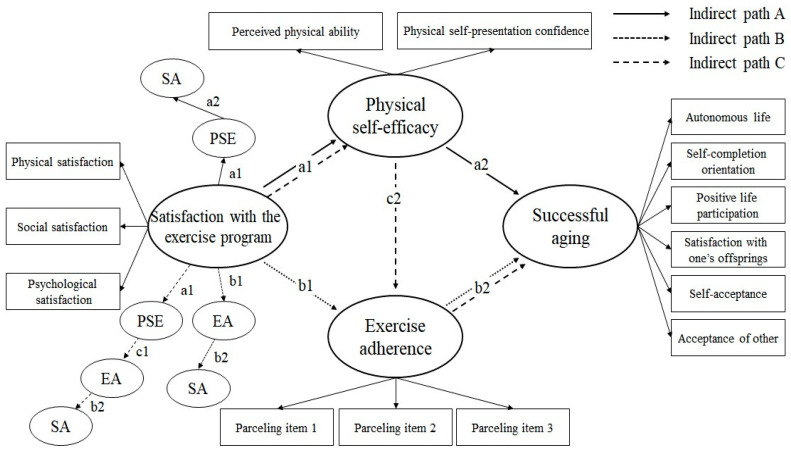
Phantom model analysis using bootstrapping. PPA, Perceived Physical Ability; PSPC, Physical Self-Presentation Confidence; SA, successful aging; PSE, physical self-efficacy; EA, exercise adherence.

The statistical significance and influence of the indirect paths were verified using phantom variables. Figure 3 shows the research model. Indirect paths referred to those paths that went through PSE and exercise adherence, which mediated the relationship between satisfaction with exercise and successful aging. There were three indirect paths: indirect paths A, B, and C. Indirect path A denoted that satisfaction with exercise affects successful aging through PSE (B = 0.019, 95% CI = 0.003, 0.056). Indirect path B denoted that satisfaction with exercise affects successful aging through exercise adherence (B = 0.045, 95% CI = 0.014, 0.192). However, indirect path C demonstrated that satisfaction with exercise does not have an indirect effect on successful aging through PSE and then exercise adherence (B = 0.021, 95% CI = −0.100, 0.019). Table 8 presents the results of analyzing these three indirect paths. Indirect paths A and B did not include zero in the 95% CI, and the two-sided significance test results were significant (*p* < 0.05). However, indirect path C was statistically insignificant based on the CI and the results of the two-sided test (*p* > 0.05). That is, satisfaction with exercise had an indirect effect on successful aging through PSE or exercise adherence but not through both. Additionally, the size of this indirect effect was very small.

## 4. Discussion

### 4.1. Interpretation of the Findings

Considering that Korea may soon become a super-aged society, discussions are ongoing on how to make Korean older adults happy. Researchers are particularly focusing on regular exercise participation as a prerequisite for successful aging. As part of these efforts, this study investigated the relationship between exercise adherence, PSE, satisfaction with exercise, and successful aging. The results of verifying the research model support those of various previous studies that have investigated the relationship between older adults’ participation in physical activities and successful aging. Several studies conducted in Korea have shown that satisfaction with exercise, PSE, exercise adherence, and successful aging are related to each other, similar to the results of this study [37,38,39,40]. The results of this study suggested that satisfaction gained by older adults from participating in regular exercise or physical activity is a crucial factor, as it directly affects PSE, exercise adherence, and successful aging. Additionally, the standardized regression coefficients of these effects were not small, and the direct impact on successful aging was found to be relatively large compared to other coefficients. These findings suggest that the satisfaction felt from participating in exercise is closely related to one’s happiness or quality of life. Similarly, numerous studies have demonstrated a relationship between exercise participation and successful aging among older adults [10,41].

Therefore, it is important to promote not only participation in regular exercise but also the physical, psychological, and social satisfaction derived from that participation. In other words, satisfaction can be increased when: (a) one’s physical health, vitality, and physical strength are improved through regular exercise; (b) there are changes in one’s sense of accomplishment, emotional stability, and self-esteem; and (c) intimacy, trust, and friendship with others are fostered. Policymakers must consider these three factors when organizing and operating exercise programs [42]. Furthermore, to promote exercise participation and increase satisfaction among the older individuals, support is needed to minimize barriers that hinder their participation. For example, even if older adults perceive exercise as enjoyable and beneficial, long-term participation may not be achieved if there is significant financial burden and pressure. A potential solution can be found in the theory of planned behavior [43]. Experts and policymakers should strive to improve older individuals’ perceptions of exercise. Efforts should be made to promote awareness of the positive benefits of exercise, and financial or instrumental support should be provided to minimize any potential obstacles that might prevent older adults from participating in exercise.

The results of this study showed that older adults’ satisfaction with exercise improves their PSE and their PSE predicts their exercise adherence. These results are very similar to those of McAuley et al. [44], who found that the use of self-control strategies by older adults participating in exercise positively affects their self-efficacy, and their self-efficacy has a positive effect on their continuance of exercise participation. In other words, the higher the self-efficacy, the higher the likelihood of older adults continuing to exercise. Furthermore, studies have consistently reported that the level of self-efficacy positively predicts exercise adherence [45,46]. Therefore, to promote older adults’ exercise continuance, it is important to focus on their PSE, and this PSE can be formed when they are sufficiently satisfied with exercise. Studies have suggested that older adults’ PSE is closely related to exercise performance [47]. In other words, older adults who believe that their body is still healthy and capable of performing physical activity are more likely to continue exercising.

Upon closer examination of the study’s results, while PSE did not have a direct effect on successful aging, it was found to mediate the relationship between satisfaction with the exercise program and successful aging. Thus, it can be interpreted that although PSE does not directly promote successful aging, it contributes to successful aging when one’s PSE increases following exercise satisfaction. The fact that PSE did not directly influence successful aging in the research model can be attributed to the characteristics of SEM, where each path’s outcome varies based on the structural relationships among all the variables involved [35]. In this model, the relationship between PSE and successful aging must consider the influence of another mediating variable: exercise adherence. Therefore, rather than interpreting PSE as having no effect on successful aging, it is more appropriate to interpret it as playing a mediating role when satisfaction with the exercise program affects successful aging. Meanwhile, exercise adherence not only had a direct effect on successful aging, but it also mediated the relationship between satisfaction with exercise and successful aging. These results showed that older adults’ satisfaction with exercise is not only directly related to their successful aging, but it also has an indirect relationship through PSE and exercise adherence. However, the indirect influence was very small compared with the direct influence. This suggests that PSE and exercise adherence play a supplementary role in the relationship between satisfaction with exercise and successful aging, and the most important task for policymakers is to increase older adults’ satisfaction with exercise.

Overall, the results of this study showed that satisfaction with exercise is very important for promoting successful aging among Korean older adults. They also showed that older adults’ satisfaction with exercise can increase their efficacy for performing physical activities and encourage them to continue exercising. This can ultimately lead them to a happy old age. Considering that successful aging is beneficial not only for older adults but also for society [8], the results of this study have salient implications for Korean society. The results of this study are expected to promote exercise participation among Korean older adults and provide useful information for policymakers and those involved in exercise programs.

### 4.2. Limitations and Directions for Future Research

This study had some limitations. Owing to the limitations of structural equation modeling, which requires model simplicity, a sub-factor analysis of the variables was not conducted. In addition, there was a lack of research analyzing the relationships between the sub-factors. Structural equation modeling explains theoretical relationships or structures, but analysis at the sub-factor level is required to identify specific relationships. Therefore, future studies should analyze the causal relationships between the sub-factors through multiple regression analysis or expansion of the research model. This study had some limitations, including the lack of control for various confounding variables that could influence the results, such as health status, socioeconomic factors, and social support. It is necessary to specifically determine whether the relationship between exercise program satisfaction and successful aging among older participants varies by sex, income level, or the type of physical activity. Furthermore, the potential impact of cultural differences and whether the model presented in this study yields different results depending on the cultural context is a topic worth exploring in future research.

It is important to note that satisfaction with exercise can vary greatly depending on the type of exercise or sport. In this study, the most popular activity among the participants was hiking. Considering that hiking is different from sports activities that include rules or competitions, the program content and satisfaction with the program can vary significantly. Therefore, it is necessary to determine whether these differences are related to satisfaction with exercise, PSE, exercise adherence, or successful aging. Lastly, this study cannot ignore the possibility of selection bias, as it focused on older adults who were participating in sports clubs or programs. Therefore, future research should include a broader sample of older adults who are not involved in such clubs. Such attempts will allow for the accumulation of in-depth insights into the healthy and happy lives of Korean older adults.

## 5. Conclusions

Among older adults who exercise regularly, satisfaction with exercise predicts PSE and exercise adherence and affects successful aging both directly and indirectly. Additionally, exercise adherence contributes to successful aging. Therefore, to promote successful aging among older adults who exercise regularly, it is important to increase their satisfaction with exercise. The results of this study are expected to be useful in promoting a happy life among Korean older adults.

## Figures and Tables

**Table 1:** Characteristics of the participants.

Characteristic	Categories	Frequency	Percentage
Sex	Male	234	63.41
	Female	135	36.59
Type of sport	Hiking	101	27.37
	Line dance	24	6.50
	Badminton	30	8.13
	Swimming	49	13.28
	Cycling	43	11.65
	Soccer	20	5.42
	Table tennis	30	8.13
	Tennis	28	7.59
	Park golf	27	7.32
	Futsal	17	4.61
Weekly frequency of participation	One day	24	6.50
	Two days	78	21.14
	Three days	108	29.27
	Four days	86	23.31
	Five days	49	13.28
	Six days	6	1.63
	Every day	18	4.88
Participation duration per session	Less than an hour	42	11.38
	Less than two hours	151	40.92
	Less than three hours	119	32.25
	Three or more hours	57	15.45
Total			369

**Table 2:** Validity and reliability of the measurement tool used for satisfaction with exercise.

Item	Estimate	Standardized Estimate	Standard Error	*t*	α
Physical satisfaction 1	1.000	0.884	-	-	0.880
Physical satisfaction 2	1.048	0.884	0.051	20.564 ***	
Physical satisfaction 3	0.850	0.762	0.049	17.180 ***	
Social satisfaction 4	1.000	0.640	-	-	0.793
Social satisfaction 5	1.339	0.905	0.108	12.356 ***	
Social satisfaction 6	1.111	0.741	0.095	11.673 ***	
Psychological satisfaction 7	1.000	0.777	-	-	0.785
Psychological satisfaction 8	1.000	0.746	0.079	12.712 ***	
Psychological satisfaction 9	1.009	0.699	0.083	12.092 ***	

*x^2^/df* = 2.978 (*x*^2^ = 71.464, *df* = 24, *p* < 0.001), comparative fit index = 0.970, Tucker–Lewis index = 0.955, standardized root mean square residual = 0.042, root mean square error of approximation = 0.073 (95% confidence interval = 0.054–0.093). *** *p* < 0.001; tested using confirmatory factor analysis and Cronbach’s *α.*

**Table 3:** Validity and reliability of the measurement tool used for exercise adherence.

Item	Estimate	Standardized Estimate	Standard Error	*t*	α
Exercise adherence 1	1.000	0.706	-	-	0.866
Exercise adherence 2	1.137	0.787	0.083	13.688 ***	
Exercise adherence 3	1.305	0.821	0.092	14.176 ***	
Exercise adherence 4	0.875	0.637	0.078	11.241 ***	
Exercise adherence 5	1.204	0.751	0.092	13.123 ***	
Exercise adherence 6	0.870	0.610	0.081	10.786 ***	

*x^2^/df* = 2.912 (*x*^2^ = 26.212, *df* = 9, *p* < 0.001), comparative fit index = 0.981, Tucker–Lewis index = 0.969, standardized root mean square residual = 0.028, root mean square error of approximation = 0.072 (95% confidence interval = 0.041–0.105). *** *p* < 0.001; tested using confirmatory factor analysis and Cronbach’s *α.*

**Table 4:** Validity and reliability of the measurement tool used for physical self-efficacy.

Item	Estimate	Standardized Estimate	Standard Error	*t*	α
PPA 1	1.000	0.811	-	-	0.928
PPA 2	0.918	0.718	0.060	15.316 ***	
PPA 3	0.829	0.709	0.055	15.043 ***	
PPA 4	1.052	0.795	0.060	17.576 ***	
PPA 5	0.761	0.681	0.053	14.275 ***	
PPA 6	0.972	0.757	0.059	16.438 ***	
PPA 7	0.997	0.774	0.059	16.940 ***	
PPA 8	0.968	0.784	0.056	17.255 ***	
PPA 9	0.978	0.745	0.060	16.299 ***	
PPA 10	0.897	0.710	0.059	15.271 ***	
PSPC 11	1.000	0.630	-	-	0.918
PSPC 12	1.034	0.696	0.089	11.629 ***	
PSPC 13	1.267	0.725	0.105	12.064 ***	
PSPC 14	1.388	0.849	0.102	13.567 ***	
PSPC 15	1.242	0.843	0.092	13.491 ***	
PSPC 16	1.254	0.743	0.102	12.312 ***	
PSPC 17	0.989	0.657	0.089	11.096 ***	
PSPC 18	1.182	0.749	0.096	12.335 ***	
PSPC 19	1.067	0.643	0.098	10.891 ***	
PSPC 20	0.897	0.597	0.088	10.238 ***	
PSPC 21	1.005	0.671	0.089	11.291 ***	
PSPC 22	0.856	0.560	0.088	9.684 ***	

*x^2^/df* = 3.066 (*x*^2^ = 628.437, *df* = 205, *p* < 0.001), comparative fit index = 0.912, Tucker–Lewis index = 0.901, standardized root mean square residual = 0.074, root mean square error of approximation = 0.075 (95% confidence interval = 0.060–0.082). PPA, Perceived Physical Ability; PSPC, Physical Self-Presentation Confidence. *** *p* < 0.001; tested using confirmatory factor analysis and Cronbach’s *α.*

**Table 5:** Validity and reliability of the measurement tool used for successful aging.

Item	Estimate	Standardized Estimate	Standard Error	*t*	α
AL 1	1.000	0.510	-	-	0.820
AL 2	1.257	0.625	0.129	9.745 ***	
AL 3	0.986	0.540	0.128	7.682 ***	
AL 4	1.027	0.621	0.122	8.403 ***	
AL 5	0.839	0.523	0.112	7.523 ***	
AL 6	0.981	0.543	0.127	7.718 ***	
AL 7	1.147	0.557	0.146	7.864 ***	
AL 8	1.361	0.665	0.157	8.688 ***	
AL 9	1.200	0.556	0.153	7.835 ***	
SCO 10	1.000	0.615	-	-	0.652
SCO 11	1.181	0.759	0.103	11.428 ***	
SCO 12	1.089	0.698	0.101	10.777 ***	
SCO 13	1.170	0.776	0.101	11.597 ***	
SCO 14	1.151	0.672	0.110	10.504 ***	
PLP 16	1.000	0.743	-	-	0.869
PLP 17	1.046	0.829	0.066	15.857 ***	
PLP 18	1.095	0.827	0.069	15.768 ***	
PLP 19	0.895	0.659	0.085	10.590 ***	
PLP 20	0.946	0.811	0.061	15.452 ***	
SWO 21	1.000	0.715	-	-	0.838
SWO 22	1.385	0.785	0.099	13.952 ***	
SWO 23	1.323	0.820	0.090	14.764 ***	
SWO 24	1.015	0.602	0.095	10.668 ***	
SWO 25	1.086	0.742	0.081	13.424 ***	
SA 26	1.000	0.809	-	-	0.842
SA 27	1.047	0.779	0.067	15.647 ***	
SA 28	0.994	0.815	0.060	16.430 ***	
AO 29	1.000	0.717	-	-	0.825
AO 30	1.230	0.833	0.087	14.154 ***	
AO 31	1.269	0.796	0.092	13.748 ***	

*x^2^/df* = 2.430 (*x*^2^ = 930.680, *df* = 383, *p* < 0.001), comparative fit index = 0.902, Tucker–Lewis index = 0.888, standardized root mean square residual = 0.052, root mean square error of approximation = 0.062 (95% confidence interval = 0.057–0.067). AL, autonomous life; SCO, self-completion orientation; PLP, positive life participation; SWO, satisfaction with one’s offspring; SA, self-acceptance; AO, acceptance of others. *** *p* < 0.001; tested using confirmatory factor analysis and Cronbach’s α.

**Table 6:** Mean, standard deviation, skewness, kurtosis, and bivariate correlation of all sub-factors.

Sub-Factor	1	2	3	4	5	6	7	8	9	10	11	12	13	14
1. Physical satisfaction	1.000													
2. Social satisfaction	0.468 ***	1.000												
3. Psychological satisfaction	0.483 ***	0.547 ***	1.000											
4. PPA	0.310 ***	0.194 ***	0.330 ***	1.000										
5. PSPC	0.243 ***	0.193 ***	0.238 ***	0.483 ***	1.000									
6. Exercise adherence M1	0.424 ***	0.441 ***	0.451 ***	0.436 ***	0.238 ***	1.000								
7. Exercise adherence M2	0.326 ***	0.353 ***	0.378 ***	0.469 ***	0.226 ***	0.721 ***	1.000							
8. Exercise adherence M3	0.308 ***	0.393 ***	0.338 ***	0.441 ***	0.167 ***	0.636 ***	0.715 ***	1.000						
9. Autonomous life	0.391 ***	0.474 ***	0.388 ***	0.191 ***	0.348 ***	0.306 ***	0.199 ***	0.211 ***	1.000					
10. Self-completion orientation	0.348 ***	0.389 ***	0.346 ***	0.256 ***	0.252 ***	0.336 ***	0.266 ***	0.238 ***	0.622 ***	1.000				
11. Positive life participation	0.398 ***	0.455 ***	0.404 ***	0.192 ***	0.229 ***	0.339 ***	0.212 ***	0.219 ***	0.679 ***	0.539 ***	1.000			
12. Satisfaction with one’s offspring	0.406 ***	0.463 ***	0.410 ***	0.085	0.238 ***	0.299 ***	0.199 ***	0.191 ***	0.610 ***	0.473 ***	0.584 ***	1.000		
13. Self-acceptance	0.359 ***	0.443 ***	0.419 ***	0.169 ***	0.267 ***	0.299 ***	0.238 ***	0.191 ***	0.622 ***	0.535 ***	0.597 ***	0.634 ***	1.000	
14. Acceptance of others	0.295 ***	0.362 ***	0.313 ***	0.281 ***	0.295 ***	0.260 ***	0.233 ***	0.225 ***	0.511 ***	0.498 ***	0.524 ***	0.564 ***	0.603 ***	1.000
Mean	4.364	4.290	4.325	3.445	3.686	4.234	4.237	4.049	4.087	4.035	4.062	4.305	4.270	3.995
Standard deviation	0.577	0.593	0.586	0.813	0.764	0.698	0.696	0.703	0.484	0.537	0.643	0.566	0.642	0.758
Skewness	−1.106	−1.024	−1.243	−0.840	−0.628	−1.208	−1.454	−1.045	−0.432	−0.572	−0.753	−1.001	−0.757	−0.343
Kurtosis	3.604	3.005	3.736	0.836	0.010	3.294	3.428	2.018	2.175	2.204	1.596	3.064	1.326	−0.257

PPA, Perceived Physical Ability; PSPC, Physical Self-Presentation Confidence. *** *p* < 0.001; tested using Pearson correlation analysis.

**Table 7:** Estimate and standardized estimate of the direct paths.

Direct Paths	Estimate	Standard Estimate	Standard Error	*t*
Satisfaction with exercise → physical self-efficacy	0.588	0.317	0.127	4.614 ***
Satisfaction with exercise → exercise adherence	0.664	0.487	0.094	7.067 ***
Satisfaction with exercise → successful aging	0.610	0.699	0.084	7.218 ***
Physical self-efficacy → exercise adherence	0.262	0.356	0.059	4.435 ***
Physical self-efficacy → successful aging	0.036	0.077	0.033	1.086
Exercise adherence → successful aging	0.121	0.190	0.056	2.158 *

* *p <* 0.05, *** *p <* 0.001; tested using structural equation modeling.

**Table 8:** Estimate of indirect paths.

Indirect Paths	Estimate	Standard Error	95% Confidence Interval	
			Lower	Upper
Path A: SWP → PSE → SA	0.019 **	0.012	0.003	0.056
Path B: SWP → EA → SA	0.081 *	0.045	0.014	0.192
Path C: SWP → PSE → EA → SA	0.021	0.032	−0.100	0.019

SWP, satisfaction with exercise; PSE, physical self-efficacy; EA, exercise adherence; SA, successful aging. * *p* < 0.05, ** *p* < 0.01; tested using structural equation modeling.

## Data Availability

The data supporting the findings of this study are available from the corresponding author upon request.
